# Final Evaluation Findings for *This Free Life*, a 3-Year, Multi-Market Tobacco Public Education Campaign for Gender and Sexual Minority Young Adults in the United States

**DOI:** 10.1093/ntr/ntab146

**Published:** 2021-07-16

**Authors:** Erik Crankshaw, Jennifer Gaber, Jamie Guillory, Laurel Curry, Matthew Farrelly, McKinley Saunders, Leah Hoffman, Ollie Ganz, Janine Delahanty, Debra Mekos, Tesfa Alexander

**Affiliations:** 1 RTI International, Center for Health Analytics, Media, and Policy, Durham, NC, USA; 2 Prime Affect Research, Dublin, Ireland; 3 Fors Marsh Group, Arlington, VA, USA; 4 Rutgers School of Public Health, Piscataway, NJ, USA; 5 Food and Drug Administration, Center for Tobacco Products, Silver Spring, MD, USA

## Abstract

**Introduction:**

*This Free Life* was the first multi-market, primarily digital campaign designed to change tobacco-related beliefs among lesbian, gay, bisexual, and transgender (LGBT) young adults. Our evaluation sought to determine whether campaign exposure resulted in changes in tobacco-related beliefs. We summarize awareness and receptivity at the conclusion of the campaign and assess the effect of campaign exposure on tobacco-related beliefs in campaign treatment markets compared with control markets.

**Aims and Methods:**

Twenty-four US designated market areas were selected to receive the campaign or serve as control markets. A baseline survey was conducted in 2016, with six follow-up surveys conducted approximately 6 months apart over the course of the 3-year campaign. 12 324 LGBT young adult survey participants were recruited via intercept interviews and social media. Campaign effects on outcomes were estimated using difference-in-difference panel regression models, with *p*-values corrected for multiple comparisons.

**Results:**

Brand and ad awareness peaked in treatment markets approximately 2.5 years into the 3-year campaign and were significantly higher in treatment than control markets. Brand equity and ad receptivity were generally high and similar across LGBT subgroups. There were small but significant campaign effects on five tobacco-related beliefs, with difference-in-difference estimates ranging from 1.9 to 5.6 percentage points.

**Conclusions:**

*This Free Life*, the first multi-market tobacco public education campaign for LGBT young adults, reached and resonated with a large and diverse population, and had a small effect on beliefs involving social aspects of smoking. These findings should inform future communication efforts aimed at reducing tobacco use among LGBT young adults.

**Implications:**

Modest overall campaign effects suggest that further research on effective campaign messaging and delivery to LGBT young adults is needed. Campaign messaging style, delivery channels, and targeted outcomes likely contributed to these findings. Health communication efforts for LGBT young adults should consider the limitations of digital media in achieving sufficient exposure. Ad style and content optimized for a digital environment is an area that will benefit from further development.

## Introduction

Cigarette smoking rates are higher in the sexual minority (eg, lesbian, gay, bisexual) adults than among their heterosexual counterparts^1,2^ and higher in transgender persons than among cisgender populations.^3^ Among the general population, tobacco public education campaigns have been effective in driving down smoking rates, but campaigns aimed at sexual and gender minority populations are rare.^4^ In 2013/2014, a campaign for lesbian, gay, bisexual, and transgender (LGBT) people in Los Angeles County, which featured a mix of online advertising and in-person outreach in bars, clubs, and gyms, raised awareness among one-third of evaluation participants and found that awareness was associated with quitting intentions.^5^ A branded social marketing campaign for LGBT young adults in Las Vegas that promoted tobacco-free partying using events and media, reported lower odds of smoking among those who understood the message and had the highest levels of campaign exposure.^6^ Several other examples of interventions geared toward the LGBT population exist but are primarily individual-focused smoking cessation programs.^7–9^ Given the high prevalence of smoking and more accepting tobacco-related norms among LGBT young adults,^10^ this population warrants an intervention focus.

The U.S. Food and Drug Administration (FDA) launched the *This Free Life* campaign in May 2016, aiming to prevent and reduce tobacco use among LGBT young adults (including those who identify as queer, pansexual, nonbinary, or another gender or sexual minority) who may smoke cigarettes occasionally but not regularly. The campaign's media agency used the social branding framework^11^ to develop the campaign strategy, which involves developing campaign brands designed to reach groups centered around a specific cultural affinity with common identities, values, norms, and beliefs.^11,12^ Social branding strategies show potential for reducing young adult smoking.^6,13,14^

*This Free Life* is unique because it is the first large-scale, LGBT-focused tobacco public education campaign. The media agency designed the *This Free Life* brand and campaign messages to couple LGBT cultural values and tobacco-free aspirations to counteract historic tobacco industry marketing to LGBT people.^15^*This Free Life* also partnered with influential LGBT community members to deliver authentic, credible campaign messages. The campaign primarily used digital and social media advertising to leverage targeting capabilities that these media offer and used additional strategies including interactive events, streaming radio, and LGBT print, and out-of-home advertising (eg, metro signage).

Preliminary evaluation findings showed that two years into the 3-year campaign, *This Free Life* had reached more than half of LGBT young adults surveyed in campaign-targeted treatment markets, with significantly higher campaign awareness in treatment markets than in control markets that had minimal campaign exposure.^16^ Perceived effectiveness (ie, ad receptivity)^17,18^ of campaign ads ranged from neutral to positive, suggesting that ads were generally well-received by the audience.^16^ Additionally, the more a respondent identified with the LGBT community, the more receptive they were to campaign advertisements.^19^ To the extent that self-reported awareness is correlated with objective measures of exposure to digital advertising,^20^ the campaign awareness and receptivity levels achieved by *This Free Life* are suggestive of early campaign implementation success. However, the minimum level of reach and receptivity required for an effective digital tobacco public education campaign remains unclear.^21^

Achieving awareness and receptivity are necessary, but not sufficient, prerequisites for an ultimately successful tobacco public education campaign.^21–23^ Our evaluation of *This Free Life* sought to determine whether campaign exposure resulted in change in tobacco-related beliefs (ie, perceptions of health consequences of smoking cigarettes, other negative effects of smoking, and social norms associated with smoking) among LGBT young adults. We assess the effect of campaign exposure on tobacco-related belief change over time in campaign-targeted treatment markets compared with control markets and explore whether the campaign had differential effects based on smoking status. We also summarize measures of *This Free Life* campaign awareness and receptivity at the conclusion of the campaign.

## Methods

### Study Design and Sample

Our evaluation of *This Free Life* used a market-level treatment-control design with one baseline survey and six follow-up surveys. To be eligible for the study, new participants were required to be age 18–24, self-identify as LGBT (including other gender and sexual minorities; see Supplementary Appendix Table 1 for study definitions of gender and sexual identity), and live in one of 24 target designated market areas (DMAs; Supplementary Appendix Table 2). We selected markets with a high prevalence of LGBT young adults who smoke occasionally; *This Free Life* advertising and events occurred in half of the DMAs, resulting in 12 treatment and 12 control markets. More details about the market selection procedure are available in Guillory et al.^16^ RTI International's Institutional Review Board approved the study.

### Data Collection

We collected baseline data from February to May 2016, prior to the May 2016 launch of the campaign, and six follow-up surveys took place at approximately 6-month intervals after campaign launch, ending July 2019. We recruited survey participants through in-person intercept methods and social media advertisements. For each follow-up survey, we invited participants who had previously completed a survey, excluding those removed from analysis due to excessive missing data, unrealistic survey completion times or response patterns, or failing attention checks. Longitudinal participation rates ranged from 22% to 41% of those invited at each follow-up. We supplemented the longitudinal sample at each follow-up with newly recruited intercept and social media participants to account for attrition. We did not conduct new social media recruitment at follow-up 4 because we recruited sufficient sample through longitudinal and intercept routes.^24^Additional information about recruitment procedures is provided in the Supplementary Materials.

## Measures

### Campaign Awareness, Receptivity, and Brand Equity

We asked participants whether they had seen the *This Free Life* logo as a measure of brand awareness at each follow-up. Responses of “Yes” were considered brand aware, and responses of “No” and “Not sure” were considered unaware. Those who had seen or heard of the brand rated 22 brand equity items on a 5-point Likert scale (1 = strongly disagree, 5 = strongly agree; see Supplementary Materials for the items). Brand equity measures the associations that an audience makes with a brand.^25^ The items comprise two constructs: brand personality/identity (Cronbach's α = 0.88), and perceived brand popularity/engagement (Cronbach's α = 0.93). Participants viewed two to four *This Free Life* video ads and were asked how frequently they had seen the video in the past 3 months (“never,” “rarely,” “sometimes,” “often,” or “very often”). Video ad awareness were ascertained at each follow-up with a different set of *This Free Life* ads from the total of seven ads that aired during the campaign. We defined overall video awareness as seeing any of the ads sometimes or more often.

We also asked participants about their reactions after viewing each ad. On a 5-point scale from strongly disagree to strongly agree, participants rated each ad on six items (eg, “This video is worth remembering,” “This video is informative”; see Supplementary Materials for the items), which together measure the perceived effectiveness of an ad (see Supplementary Appendix Table 3 for Cronbach's alphas). The average score across these items for each ad was calculated for each participant.

### Belief Outcomes

The main outcomes of the campaign evaluation were tobacco-related belief questions—most of which were measured as scales and developed specifically for this evaluation to measure campaign priority beliefs. Outcomes include perceptions of negative health and social consequences and perceived social norms associated with smoking cigarettes. They also include measures of perceived ability to avoid smoking cigarettes in various social situations and, among current smokers, measures of desire and motivation to quit. Response options for most belief questions were on a Likert-type scale (eg, 1 = Strongly disagree, 5 = Strongly agree). Exploratory factor analyses confirmed that these were unidimensional scales, and we estimated scale reliability (Cronbach's Alpha) for each of 13 scales for each survey. Scales that had alpha estimates greater than 0.7 at each follow-up were considered to have high reliability and were used as outcomes in the evaluation models, resulting in nine scales (Supplementary Appendix Table 4). We calculated an average across all relevant items in a scale to create a score for each participant.

All individual belief items, including six that were not included in scales, were dichotomized for analyses to aid in interpretability and focus on the primary category of interest (noted in bold in Supplementary Appendix Table 4).

### Covariates

Fixed effects panel regression models controlled for age (in years), employment status (full-time, part-time, do not work), education (high school or less, some college, college plus), student status (student, non-student), LGBT identity (cisgender female lesbian or gay, cisgender male gay, cisgender female bisexual, cisgender male bisexual, gender minorities, cisgender other sexual identity), media use, smoking status, and scales measuring LGBT community involvement and connection^19^ with items adapted from the LGBT Identity Affirmation scale,^26^ LGBT Identity Centrality scale,^26^ and Identification with the LGBT Community scale^27,28^ (see Supplementary Materials for more detailed covariate definitions). Interaction terms were included for age by employment and age by education as employment and education are dependent on age during young adulthood. An interaction of treatment group by round of data collection was the primary measure of campaign exposure and predictor of interest. Random effects regression models controlled for additional time-invariant covariates: recruitment source (intercept or social media) and race/ethnicity (non-Hispanic White, non-Hispanic Black, Hispanic, non-Hispanic other).

### Statistical Analysis

We used descriptive statistics to summarize sample characteristics, self-reported awareness of the *This Free Life* brand, awareness of any video ad, perceived effectiveness scale scores for individual video ads, and brand equity at each round.

We estimated the campaign treatment effect on all scales and individual outcomes (both those included in scales and those not included in scales) using difference-in-difference panel regression models.^29^ The panel linear regression models for scale outcomes were fixed effects models that accounted for omitted variable bias; we conducted robust Hausman tests, which confirmed the need for fixed rather than random effects. Panel logistic regression models for dichotomized individual outcomes with unbalanced panel designs are not accommodated with fixed effects, so these were random effects models.

The main predictor in all models was the difference-in-difference interaction between treatment and round of data collection (relative to pre-campaign baseline data collection) to determine whether changes in outcomes throughout the campaign were greater in treatment than control markets. We included the covariates defined earlier in all models. We used post-estimation margins commands in Stata version 15 to estimate predicted average scores (for scale outcomes), probabilities (for dichotomized outcomes), and contrasts in the change from baseline to each follow-up between treatment and control.

We also assessed differential campaign effects among smoking status subgroups since the smoking status was a significant covariate in all primary models, suggesting important subgroup differences. This additional analysis was accommodated by adding a 3-way interaction term between treatment, round of data collection, and smoking status. We used Stata's margins commands to estimate predicted average scores and contrasts for each smoking status subgroup by treatment and round of data collection.

All panel regression models accounted for clustering due to repeated measures at the participant level using XT settings in Stata. Stata's XT suite is robust for unbalanced panel designs.^30^ All *p*-values were corrected for multiple comparisons. We used the Benjamini and Hochberg^31^ method of controlling the false discovery rate (FDR), as this approach has more statistical power in cases with many statistical significance tests than methods aimed at controlling for the Familywise Error Rate (eg, Bonferroni). We selected an FDR of 20% to minimize the likelihood of falsely rejecting a true hypothesis under the rationale that identifying potential campaign impacts would benefit future campaign planners.^31^

## Results

### Sample Descriptive Statistics

We report sample characteristics in Table 1. The sample sizes at each round of data collection ranged from 2788 to 4177, and a total sample of 12 324 LGBT young adults participated in at least one wave of the study and were included in these analyses. Participants aged 21+ made up 72%–83% of the sample at each round. Cisgender gay men were the most common gender and sexual identity (30%–45%), followed by bisexual women (16%–23%), cisgender lesbian women (17%–22%), gender minorities (8%–19%), bisexual men (4%–5%), and other sexual minorities (4%–5%). White non-Hispanic participants made up less than or equal to half of each sample (38%–50%). Most participants were not current students (54%–64%), and a large majority of each sample reported having some level of college education (74%–80%). Nearly half of respondents in each sample were employed full-time. We saw shifts in sample demographics from baseline to follow-up 6 including increasing average age, increasing proportion of gender minorities, decreasing proportion of cisgender gay males, and increasing education. Ever but not current smokers made up 34%–41% of the sample of LGBT young adults at each wave, followed by non-daily smokers (22%–34%), never smokers (20%–31%), and daily smokers (5%–11%). Prevalence of any past-30-day cigarette smoking ranged from 28% to 47% of each sample. These demographic shifts over time are due to both changes within the longitudinal sample and differences in samples recruited at each follow-up.

**Table 1.** Demographic and Psychographic Characteristics of Samples at Each Round

Demographic characteristic	Baseline (4035^a^)	Follow-up 1 (2788^a^)	Follow-up 2 (3548^a^)	Follow-up 3 (4177^a^)	Follow-up 4 (3893^a^)	Follow-up 5 (4134^a^)	Follow-up 6 (4071^a^)
**Age**							
18	345 (9%)	131 (5%)	149 (4%)	189 (5%)	120 (3%)	140 (3%)	128 (3%)
19	375 (9%)	222 (8%)	315 (9%)	310 (7%)	252 (6%)	267 (6%)	242 (6%)
20	396 (10%)	218 (8%)	316 (9%)	373 (9%)	332 (9%)	334 (8%)	328 (8%)
21	776 (19%)	474 (17%)	472 (13%)	521 (12%)	422 (11%)	512 (12%)	491 (12%)
22	640 (16%)	563 (20%)	675 (19%)	721 (17%)	540 (14%)	561 (14%)	568 (14%)
23	783 (19%)	582 (21%)	684 (19%)	786 (19%)	723 (19%)	713 (17%)	640 (16%)
24	720 (18%)	598 (21%)	663 (19%)	777 (19%)	768 (20%)	779 (19%)	748 (18%)
25	—	—	274 (8%)	389 (9%)	495 (13%)	520 (13%)	563 (14%)
26	—	—	—	111 (3%)	241 (6%)	308 (7%)	363 (9%)
**Gender & sexual identity**							
Cisgender female lesbian or gay	876 (22%)	479 (17%)	669 (19%)	787 (19%)	726 (19%)	778 (19%)	708 (17%)
Cisgender male gay	1811 (45%)	1218 (44%)	1353 (38%)	1400 (34%)	1349 (35%)	1299 (31%)	1237 (30%)
Cisgender female bisexual	637 (16%)	436 (16%)	662 (19%)	865 (21%)	778 (20%)	888 (21%)	952 (23%)
Cisgender male bisexual	215 (5%)	152 (5%)	178 (5%)	201 (5%)	164 (4%)	188 (5%)	180 (4%)
Gender minorities	339 (8%)	384 (14%)	529 (15%)	722 (17%)	688 (18%)	766 (19%)	782 (19%)
Cisgender other sexual minority	150 (4%)	107 (4%)	140 (4%)	188 (5%)	180 (5%)	194 (5%)	201 (5%)
**Race/ethnicity**							
White, non-Hispanic	1856 (46%)	1315 (47%)	1705 (48%)	2098 (50%)	1478 (38%)	2074 (50%)	2037 (50%)
Black, non-Hispanic	403 (10%)	277 (10%)	349 (10%)	393 (9%)	255 (7%)	355 (9%)	346 (9%)
Hispanic	1065 (26%)	714 (26%)	841 (24%)	941 (23%)	973 (25%)	986 (24%)	971 (24%)
American Indian or Alaska Native	26 (1%)	11 (0%)	20 (1%)	20 (0%)	12 (0%)	17 (0%)	13 (0%)
Asian or Pacific Islander	151 (4%)	117 (4%)	148 (4%)	161 (4%)	117 (3%)	149 (4%)	151 (4%)
Multiracial	391 (10%)	287 (10%)	391 (11%)	475 (11%)	324 (8%)	476 (12%)	461 (11%)
Other, non-Hispanic	82 (2%)	50 (2%)	66 (2%)	64 (2%)	58 (1%)	56 (1%)	65 (2%)
**Education**							
High school or less	1040 (26%)	626 (22%)	792 (22%)	921 (22%)	766 (20%)	842 (20%)	847 (21%)
Some college	2049 (51%)	1382 (50%)	1676 (47%)	1966 (47%)	1746 (45%)	1940 (47%)	1831 (45%)
College +	887 (22%)	773 (28%)	1069 (30%)	1273 (30%)	1369 (35%)	1346 (33%)	1386 (34%)
**Current student**							
Yes	1746 (43%)	1145 (41%)	1476 (42%)	1624 (39%)	1450 (37%)	1509 (37%)	1427 (35%)
No	2183 (54%)	1590 (57%)	2014 (57%)	2483 (59%)	2394 (61%)	2557 (62%)	2591 (64%)
**Employment**							
Full-time	1653 (41%)	1278 (46%)	1611 (45%)	1960 (47%)	2037 (52%)	2024 (49%)	2172 (53%)
Part-time	1558 (38%)	1024 (37%)	1297 (37%)	1457 (35%)	1283 (33%)	1412 (34%)	1323 (33%)
Don't currently work	722 (18%)	438 (16%)	570 (16%)	670 (16%)	514 (13%)	643 (16%)	518 (13%)
**Smoking status**							
Never smokers	811 (20%)	691 (25%)	915 (26%)	1111 (27%)	1084 (28%)	1150 (28%)	1243 (31%)
Ever, not current, smokers	1360 (34%)	1008 (36%)	1279 (36%)	1535 (37%)	1561 (40%)	1691 (41%)	1644 (41%)
Non-daily phantom smokers^b^	970 (24%)	610 (22%)	772 (22%)	843 (20%)	713 (18%)	739 (18%)	680 (17%)
Non-daily smokers^b^	386 (10%)	208 (8%)	261 (7%)	296 (7%)	241 (6%)	273 (6%)	215 (5%)
Daily smokers	445 (11%)	248 (9%)	291 (8%)	360 (9%)	256 (7%)	249 (6%)	217 (5%)

^a^Numbers may not add up to total *N* at each wave due to missing respondent data (which was excluded from denominators in percent calculations).

^b^Bullseye target audience is composed of LGBT young adults who are non-daily smokers.

At baseline, treatment and control groups (*N* = 2413 and 1622, respectively) exhibited unadjusted demographic differences. Chi-square tests revealed significant differences among the distributions of gender and sexual identity, race/ethnicity, education, smoking status, and recruitment source. Compared with the control group, the treatment group had a smaller proportion of gender minorities; a larger proportion of cisgender gay males; a larger proportion of Hispanic respondents; a larger proportion of those with greater than a college education; a larger proportion of ever but not current smokers; a smaller proportion of daily smokers; and a larger proportion of those recruited through social media.

### *This Free Life* Awareness, Receptivity, & Brand Equity

**Figure 1.**
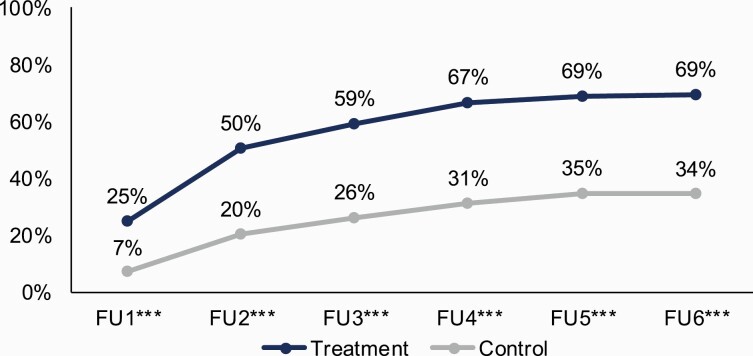
*This Free Life* brand awareness by evaluation market type over time. FU = Follow-up. ***p < .001, denotes difference between respondents in treatment and control markets.

Consistent with mid-campaign evaluation results previously reported,^16^*This Free Life* brand awareness was higher in treatment than control markets (*p* < .001) at every follow-up (Figure 1). At follow-up 5, brand awareness peaked among treatment and control markets at 69% and 35%, respectively. Brand awareness in treatment markets was consistently highest among gender minorities (peaking at 78%) and lowest among bisexual men (peaking at 53%) (*p* <.001). At the end of the campaign, respondents over age 20 (71%; *p* < .001) and those who were ever but not current smokers (73%; *p* < .01) reported significantly higher awareness of *This Free Life* than other age and smoking categories. Overall video ad awareness was higher in treatment than control markets (*p* < .001) at every follow-up (data not shown). Awareness of any video ad among respondents in treatment markets peaked at 60% at follow-up 5 and dropped to 48% at follow-up 6; this was due to the exclusion of the ad with the highest overall awareness (*Flawless*) from the final survey. Video ad awareness among control markets peaked at 31%. Among treatment respondents, the increase in overall video awareness from follow-ups 1 to 6 was significant (*p* < .001) but not among control respondents. By the end of the campaign, there were no differences in video ad awareness by LGBT status, age, or smoking status subgroups. Supplementary Appendix Table 3 provides *This Free Life* video ad perceived effectiveness scores from follow-ups 1 to 6. Perceived effectiveness scores for *This Free Life* ads ranged from 3.30 to 3.94 across survey rounds.

*This Free Life* brand equity scores for brand personality and identity (eg, “This Free Life is motivating”) were consistently higher than for brand popularity and engagement (eg, “I’d wear a This Free Life t-shirt”). At follow-up 6, brand personality and identity scores were high for all gender and sexual identity subgroups, but they were highest among cisgender lesbian women (4.05) and cisgender other sexual minorities (4.06) and lowest among gender minorities (3.90). Brand personality and identity scores were also highest among never smokers (4.10) and ever but not current smokers (4.04). Brand popularity and engagement scores were lower overall, but among gender and sexual identity subgroups, cisgender gay men reported the highest levels (3.13), and gender minorities reported the lowest (2.98). Brand popularity and engagement did not differ by age or smoking subgroups.

### Panel Regression Difference-in-Difference Models

Of the nine belief scales, two related to social perceptions of smoking (perceived positive attributes of tobacco-free people scale, and perceived ability to avoid smoking in social situations scale) showed small campaign effects (Table 2), but after correcting for multiple comparisons, significance was lost. See Supplementary Appendix Table 5 for results for scales that did not have significant effects. We conducted subgroup analyses by smoking status for all belief scales. Several small interaction effects were initially estimated, but all results by smoking status lost significance after correcting for multiple comparisons and therefore may be due to chance (Supplementary Appendix Table 6).

**Table 2.** Knowledge, Attitude, and Belief (KAB) Scales and Items with Significant Effects Before Correction

Outcome	Follow-up round	*N* ^a^	DiD estimate [95% CI]^b^	*p*	Benjamini–Hochberg corrected significance^c^
Perceived positive attributes of tobacco-free people scale	Follow-up 4	11 667	0.095 [0.018, 0.173]	.01	Not significant
Perceived ability to avoid smoking in social situations scale	Follow-up 6	11 677	0.107 [0.014, 0.199]	.02	Not significant
Would you hang out with someone who smokes cigarettes?	Follow-up 2 Follow-up 3 Follow-up 4 Follow-up 6	11 643	0.016 [0.002, 0.030] 0.019 [0.006, 0.032] 0.023 [0.009, 0.037] 0.025 [0.010, 0.040]	.03 <.01 <.01 <.01	Not significant Significant Significant Significant
People who are tobacco-free are attractive.	Follow-up 2 Follow-up 3 Follow-up 4 Follow-up 5	11 654	0.053 [0.017, 0.088] 0.037 [0.002, 0.072] 0.056 [0.020, 0.093] 0.051 [0.016, 0.087]	<.01 .04 <.01 <.01	Significant Not significant Significant Significant
People who are tobacco-free are trendsetting.	Follow-up 1 Follow-up 4	11 643	0.035 [0.012, 0.058] 0.029 [0.007, 0.051]	<.01 .01	Significant Not significant
Using tobacco makes life harder.	Follow-up 1	11 652	0.053 [0.016, 0.090]	<.01	Significant
How sure are you that, if you really wanted to, you could avoid smoking cigarettes if you are at a party, bar, or club?	Follow-up 3 Follow-up 6	11 669	0.043 [0.007, 0.080] 0.051 [0.014, 0.087]	.02 <.01	Not significant Significant
People who are tobacco-free are confident.	Follow-up 2 Follow-up 4	11 653	0.030 [0.002, 0.057] 0.037 [0.009, 0.065]	.03 .01	Not significant Not significant
According to most people who hang out where I hang out, it is very important for me to not smoke cigarettes.	Follow-up 4	11 654	0.040 [0.008, 0.072]	.01	Not significant
People who are tobacco-free are happy.	Follow-up 6	11 653	0.038 [0.007, 0.068]	.02	Not significant
According to people my age in LGBT communities, it is very important for me to not smoke cigarettes.	Follow-up 3	11 649	0.035 [0.007, 0.064]	.02	Not significant
Would you dance with someone who smokes cigarettes?	Follow-up 1 Follow-up 3 Follow-up 4 Follow-up 6	11 629	0.024 [0.004, 0.044] 0.022 [0.003, 0.042] 0.025 [0.005, 0.046] 0.024 [0.003, 0.046]	.02 .03 .02 .03	Not significant Not significant Not significant Not significant
Would you kiss someone who smokes cigarettes?	Follow-up 3	11 630	0.034 [0.006, 0.063]	.02	Not significant
How worried are you that smoking will damage your physical appearance or attractiveness?	Follow-up 4	5256	0.072 [0.012, 0.132]	.02	Not significant
If I started to smoke occasionally, I would not become addicted.	Follow-up 5	11 660	0.039 [0.003, 0.074]	.03	Not significant

^a^ *N* is the number of unique respondents in each model, with between 1 and 7 observations per respondent.

^b^Difference-in-differences (DiD) estimate is the contrast between the change in predicted probabilities from baseline to a given follow-up round for Treatment vs. Control. In cases where the outcome had no significant results, the range of DiD estimates (and corresponding *p*-values) is reported in Supplementary Appendix Table 7.

^c^All *p*-values were adjusted for multiple comparisons using a false discovery rate of 20%.

Control variables in logistic regression models with random effects were age, education, employment status, student status, smoking status, LGBT identity, race/ethnicity, recruitment source, and scales for media use, LGBT involvement, and LGBT connection.

CI = confidence interval; LGBT = lesbian, gay, bisexual, and transgender.

**Figure 2.**
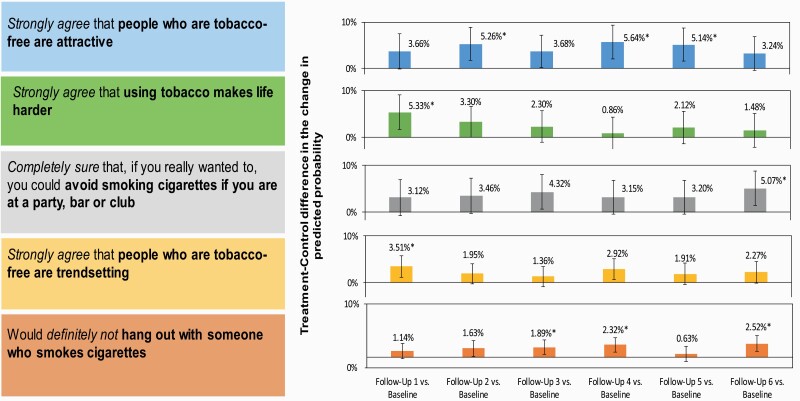
Belief outcomes with significant campaign treatment effects. * Denotes effects that remained significant after correcting for multiple comparisons.

To evaluate whether the campaign influenced specific beliefs, we ran panel logistic regression models for the individual dichotomized outcomes. Out of 36 individual outcomes, 13 items had small campaign effects for at least one follow-up (Table 2), and after correcting for the FDR, five results remained significant: (1) people who are tobacco-free are attractive (strongly agree), (2) using tobacco makes life harder (strongly agree), (3) ability to avoid smoking when at a party, bar, or club where most people are smoking (completely sure), (4) people who are tobacco-free are trendsetting (strongly agree), and (5) willingness to hang out with someone who smokes cigarettes (definitely not). Here, we present results for these five individual outcomes (Figure 2). See Supplementary Appendix Table 7 for non-significant results.

Figure 2 shows that the predicted probability of respondents strongly agreeing that “people who are tobacco-free are attractive” increased from baseline by 5.6 percentage points more for treatment than control at follow-up 4 [95% CI: 2.0–9.3] (ie, treatment-control difference-in-difference); the treatment-control difference-in-difference from baseline was also significant at follow-ups 2 and 5 for this item. The predicted probability of respondents strongly agreeing that “using tobacco makes life harder” increased from baseline by 5.3 percentage points more among treatment than control at follow-up 1 [95% CI: 1.6–9.0]. The predicted probability of respondents feeling “completely sure” that they “could avoid smoking if [they] are at a party, bar, or club where most people are smoking” increased from baseline by 5.1 percentage points more among treatment than control at follow-up 6 [95% CI: 1.4–8.7]. The predicted probability of respondents strongly agreeing that “people who are tobacco-free are trendsetting” increased from baseline by 3.5 percentage points more for treatment than control at follow-up 1 [95% CI: 1.2–5.8]. The predicted probability of participants responding that they would definitely not “hang out with someone who smokes cigarettes” increased from baseline to follow-up 6 by 2.5 percentage points more in treatment than control [95% CI: 1.0–4.0]; the treatment-control difference-in-difference from baseline was also significant at follow-ups 3 and 4.

## Discussion

This evaluation of FDA's *This Free Life*, the first large-scale tobacco public education campaign for LGBT young adults, sought to determine if the campaign impacted smoking-related beliefs during the 3-year campaign. The primarily digital campaign achieved reasonably high levels of brand and ad awareness in treatment markets, while minimizing exposure in campaign markets, which facilitated a rigorous evaluation design. An assessment of ad receptivity (perceived ad effectiveness) and brand equity suggests that the brand and most of the campaign ads resonated with the audience. Campaign brand awareness, brand equity, ad awareness, and ad receptivity were generally similar across LGBT subgroups, speaking to the broad appeal of the campaign.

Some LGBT subgroup differences in campaign awareness and receptivity are worth noting. We found that although brand and ad awareness were relatively high for gender minorities, brand equity was generally lower for this group. Evaluation findings from early in the campaign found similar patterns for awareness and receptivity among gender minorities.^19^ In a recent qualitative study with transgender and gender diverse young adults, Hinds et al.^32^ found that overall perceptions of *This Free Life* were positive, with participants noting that the combination of visible transgender representation and positive tone was affirming. But the focus in several of the ads on physical appearance and bar/club culture was viewed negatively by some, and as reflecting a perceived focus on cisgender gay males.^32^*This Free Life* aimed to appeal broadly to LGBT young adults; our results suggest that it was largely successful in this regard, but future targeted campaigns may need to balance the desire for broad appeal with the significant diversity of the LGBT population.

Our findings suggest that *This Free Life* may have had a small impact on a subset of beliefs related to social aspects of smoking. This is tempered by the fact that due to the multiple statistical comparisons we conducted, the possibility that significant findings are due to chance cannot be discounted. After correcting for the FDR, 5 of 45 outcomes assessed remained significant, though the size of the effects was modest. Each of these five outcomes were measures of social aspects of smoking, including positive perceptions about tobacco-free people, and desire and perceived ability to avoid smokers/smoking in social situations. There were no campaign effects on beliefs about negative health consequences of occasional smoking, a secondary campaign objective.

We posit several reasons for modest campaign effects. As a primarily digital campaign with no broadcast presence, *This Free Life* may not have had sufficient reach and frequency of exposure in the treatment market. Campaign awareness peaked at approximately 60% in treatment markets; the Centers for Disease Control & Prevention (CDC) has estimated that campaign advertisements need to reach 75%–85% of the target audience to produce population-level impacts.^21^ This oft-cited guidance is based on evaluations of state and national campaigns that included broadcast as the primary campaign delivery source—it has not yet been established that a digital campaign can achieve similar levels of reach in an increasingly crowded media landscape.

This campaign developed digital video ads similar to traditional broadcast ads in terms of length (ie, 30 s) and a variety of other digital content. It is possible that audiences interact with digital ads differently than broadcast ads, and that digital ads may need to be designed to more rapidly capture attention and engage audiences, perhaps with shorter-length consistent messaging delivered via a variety of creative styles and visually arresting imagery. Digital advertising remains an area that could benefit from further research and development.

*This Free Life* was developed under a social branding framework that sought to link LGBT cultural norms and aspirations with a tobacco-free lifestyle, and generally used a positive, affirming style to deliver messages to build brand identity and authenticity. Although the campaign brand and messages were well received by the target population, this approach represents a departure from successful antismoking campaigns that have employed hard-hitting, negative-emotional messaging.^21^ The positive messaging was relatively well-liked by the audience, but may have been less memorable and impactful than messaging explicitly designed to produce negative emotional responses. It is unclear what role if any this distinction in messaging style may have had, and future research on effective messaging with this population is warranted.

The “social branding” style and content of this campaign represents a novel approach that has shown promise in previous campaigns designed for young adult peer crowds (eg, LGBT young adults, partiers),^6,13,14^ although these campaigns were local, single-market campaigns with significant community-level involvement and engagement. It is plausible that the social branding approach may require a level of community engagement that would be challenging to achieve in a large-scale, semi-national campaign.

*This Free Life* was designed to appeal to a wide audience, with messages intended to be relevant to non- and former-smokers, current non-daily smokers, and daily smokers, but the primary focus was LGBT young adults who smoke occasionally or socially, with a goal of preventing the transition from occasional smoking to daily smoking. Unlike youth smoking prevention or adult smoking cessation campaigns, this focus does not lend itself to a clear call-to-action (eg, “quit smoking”), which has been a hallmark of effective prevention and cessation messaging. This lack of a clear call-to-action, coupled with many tobacco-related beliefs included in the campaign messages, likely diluted the potential impact of the campaign.

Although this evaluation benefitted from a rigorous design, there are important limitations to consider. The large number of tobacco-related belief outcomes and the issue of multiple statistical comparisons bears repeating. Even with the 20% FDR correction, the possibility that the reported results are due to chance cannot be fully discounted. Future campaigns could focus on a small, well-defined number of messaging targets and campaign objectives to maximize campaign impact and the ability of the evaluation to detect effects.

This study used a combination of in-person and social media recruitment, enabling us to sample a large, diverse LGBT young adult population, but this approach limits the generalizability of these results. The in-person recruitment, which occurred in bars, nightclubs, and other social settings in cities likely resulted in a sample that is more urban and potentially more socially active than the general population. Rural and socially isolated LGBT young adults may be under-represented.

Our treatment and control markets were not randomly assigned, and although the difference-in-difference analytic approach we used largely controls for market-level fixed (time invariant) effects, we cannot fully account for changes in markets (eg, tobacco control policies, local media campaigns) that may have differentially impacted treatment or control markets.

We recruited new survey participants at each follow-up, resulting in a sample with both longitudinal and cross-sectional respondents. The treatment and control samples were recruited with identical procedures and had similar retention rates, and our primary analytic approach (difference-in-difference panel regression models adjusted for clustering at the individual level) is well-suited for unbalanced samples like this. Despite these strengths, we cannot fully discount the possibility that unmeasured differences between longitudinal and cross-sectional respondents may confound these findings.

*This Free Life*, the first multi-market, primarily digital tobacco public education campaign to directly target LGBT young adults, reached and resonated with a large and diverse population, and despite the noted challenges had a potential small effect on certain attitudes about the social aspects of smoking. Findings from the *This Free Life* campaign, which ended in 2020, have informed continued FDA efforts to reach LGBTQ+ audiences and deliver tobacco education messaging and lessons learned from this evaluation could inform future media campaigns aimed at reducing tobacco use among LGBT young adults.

## Supplementary Material

A Contributorship Form detailing each author’s specific involvement with this content, as well as any supplementary data, are available online at https://academic.oup.com/ntr.

ntab146_suppl_Supplementary_Materials

ntab146_suppl_Supplementary_Taxonomy_Form

## Data Availability

The data underlying this article cannot be shared publicly due to the need to protect the privacy of individuals that participated in the study. Anonymized data will be shared on reasonable request to the corresponding author with permission of FDA’s Center for Tobacco Products.
