# Supplementary material for: Final Evaluation Findings for This Free Life, a 3-Year, Multi-Market Tobacco Public Education Campaign for Gender and Sexual Minority Young Adults in the United States
Source: Nicotine Tob Res. 2021 Jul 16;24(1):109–17. doi: 10.1093/ntr/ntab146 (PMC8666114; doi:10.1093/ntr/ntab146)
Supplement: ntab146_suppl_Supplementary_Materials [file ntab146_suppl_supplementary_materials.docx]

# Supplementary Materials

Appendix Table 1. Study Definitions of Gender and Sexual Identity

| Category Label | Category Definition |
| --- | --- |
| Lesbian or Gay Cisgender Women | - “Female” to “What sex were you assigned at birth, on your original birth certificate?” - “Female” to “What is your current gender identity?” - “Lesbian or Gay” to “Which of the following best represents how you think of yourself?” |
| Gay Cisgender Men | - “Male” to “What sex were you assigned at birth, on your original birth certificate?” - “Male” to “What is your current gender identity?” - “Gay” to “Which of the following best represents how you think of yourself?” |
| Bisexual Cisgender Women | - “Female” to “What sex were you assigned at birth, on your original birth certificate?” - “Female” to “What is your current gender identity?” - “Bisexual” to “Which of the following best represents how you think of yourself?” |
| Bisexual Cisgender Men | - “Male” to “What sex were you assigned at birth, on your original birth certificate?” - “Male” to “What is your current gender identity?” - “Bisexual” to “Which of the following best represents how you think of yourself?” |
| Gender Minorities | - “Trans male/Trans man,” “Trans female/Trans woman,” “Genderqueer/Gender non-conforming,” or “Different identity,” to “What is your current gender identity?” OR - “Something else” to “Which of the following best represents how you think of yourself?” and “You are transgender, transsexual, or gender variant” to “What do you mean by something else?” and “What is your current gender identity” is not “female,” “male,” or “prefer not to answer.” OR - “Male” to “What sex were you assigned at birth, on your original birth certificate?” and “female” to “What is your current gender identity?” OR - “Female” to “What sex were you assigned at birth, on your original birth certificate?” and “male” to “What is your current gender identity?” |
| Other Sexual Identity, Cisgender | - Cisgender male (“Male” to “What sex were you assigned at birth, on your original birth certificate?” and “Male” to “What is your current gender identity?”) OR - Cisgender female (“Female” to “What sex were you assigned at birth, on your original birth certificate?” and “Female” to “What is your current gender identity?”) AND - “Something else” to “Which of the following best represents how you think of yourself?” OR - “Something else” to “Which of the following best represents how you think of yourself?” and “You are not straight, but identify with another label such as queer, tri-sexual, omnisexual, or pansexual,” “You have not figured out or are in the process of figuring out your sexuality,” “You do not think of yourself as having sexuality,” “You do not use labels to identify yourself,” or “You mean something else,” to “What do you mean by something else?” OR - “I don’t know the answer” “Which of the following best represents how you think of yourself?” and “You understand the words, but you have not figured out or are in the process of figuring out your sexuality,” “You mean something else” to “What do you mean by don’t know?” |

Appendix Table 2. Target Designated Market Areas

| Treatment | Control |
| --- | --- |
| Atlanta, GA | Austin, TX |
| Boston (Manchester), MA | Denver, CO |
| Chicago, IL | Detroit, MI |
| Dallas-Ft. Worth, TX | Norfolk/Newport News, VA |
| Los Angeles, CA | Phoenix-Prescott, AZ |
| Miami-Ft. Lauderdale, FL | Pittsburgh, PA |
| Minneapolis-St. Paul, MN | Providence, RI-New Bedford, MA |
| New York, NY | Sacramento-Stockton-Modesto, CA |
| Portland, OR | San Antonio, TX |
| San Diego, CA | Seattle-Tacoma, WA |
| San Francisco-Oakland-San Jose, CA | St. Louis, MO |
| Washington, DC–Hagerstown, MD | Tampa-St. Pete-Sarasota, FL |

## Sample Recruitment Procedures

### Intercept Recruitment

We recruited participants via in-person intercept in social venues (e.g., bars, nightclubs) identified a priori as popular among LGBT young adults in the 24 evaluation markets. Additional recruitment took place at LGBT events, Pride festivals, and other festivals. Once an appropriate social venue was identified, field interviewers obtained permission from venue management to recruit young adults to complete screeners. Field interviewers approached young adults who appeared to be within the target age range and asked them to complete a brief screener for $10 cash. We sent eligible participants a personalized link to the survey via e-mail or short message service (SMS) text message through the survey software Qualtrics. We sent up to three reminder messages. Those who completed the survey received $20, with an additional $5 for participants who completed the survey within 48 hours of receiving the invitation.

### Social Media Recruitment

We also recruited LGBT young adults through social media advertising campaigns on Facebook and Instagram. Although we used social media ads to recruit members from all LGBT subgroups, targeted ads were useful for recruiting transgender and other gender and sexual minorities who were more difficult to reach via intercept. Individuals who clicked on the ads were directed to a screener. Eligible young adults were routed directly to the full survey and received a $20 upon completion. More details on recruitment are available in Guillory et al., 2018.^24^

### Longitudinal Recruitment

For each follow-up survey after baseline, we invited individuals who had previously completed a survey at any round to participate again. Beginning with follow-up 2, the age eligibility for longitudinal respondents was extended to age 26 to allow 24-year-old baseline respondents to remain in the study. We sent a survey link through e-mail and SMS text message, with up to three reminders. Former participants logged in using the e-mail address they provided when they entered the study before proceeding to eligibility questions. Participants who were no longer eligible screened out of the study. We provided $20 to those who were eligible and proceeded to complete the survey, with an additional $5 for survey completion within 48 hours of receiving the invitation.

## Study Measures of Brand Equity

We measured brand equity using 22 items. Respondents rated their agreement with each item on a 5‑point Likert scale (strongly disagree, disagree, neither agree nor disagree, agree, strongly agree). Using factor analysis, two brand equity sub-scales were created: a brand popularity and engagement scale, and a brand personality and identity scale.

The first 13 items used to assess brand popularity and engagement were the following (Cronbach’s α = 0.93):

- I want to help promote *This Free Life.*
- I’d defend *This Free Life* if someone was making fun of it.
- I’d wear a *This Free Life* T-shirt.
- I talk to my friends about *This Free Life.*
- If I had the chance, I would tell people I know to watch *This Free Life* videos.
- If I see anything from *This Free Life*, I check it out.
- *This Free Life* is popular with people like me.
- People are talking about *This Free Life.*
- *This Free Life* videos are becoming more popular with the people who hang out where I hang out.
- *This Free Life* videos are for people like me.
- If I had the chance, I would tell people I know to check out *This Free Life* events.
- Checking out *This Free Life* at events is becoming more popular with the people who hang out where I hang out.
- *This Free Life* events are for people like me.

The eight items measuring brand personality and identity asked respondents to what extent they agreed with each of the following (Cronbach’s α = 0.88):

*“This Free Life* is…”

- Trendsetting
- Motivating
- Confident
- Outgoing
- Always looks good

As well as “When I think of *This Free Life*, I think…”

- I have the freedom to be tobacco-free.
- Using tobacco can undo some of the progress I have made.
- Tobacco use is not attractive.

## Study Measures of Ad Receptivity (Perceived Effectiveness)

We asked respondents about their reactions after viewing each video ad. On a 5‑point scale from strongly disagree to strongly agree, respondents rated each ad on the following six items, which together measure the perceived effectiveness or receptivity of an ad:

- “This video is worth remembering,”
- “This video grabbed my attention,”
- “This video is powerful,”
- “This video is informative,”
- “This video is meaningful to me,” and
- “This video is convincing.”

In addition, one item asked respondents to indicate “whether the video made using tobacco look like something you would or wouldn’t want to do” on a scale of 1 to 5, where 1 = ”The video makes me want to use tobacco” and 5 = ”The video makes me want to not use tobacco.”

Appendix Table 3. Perceived Effectiveness (PE) of *This Free Life* Video Advertisements, Follow-Ups 1 through Follow-Up 6

| Video Advertisement | PE Scale Cronbach’s α | Mean^a^ | SD |
| --- | --- | --- | --- |
| Follow-Up 1 |  |  |  |
| Flawless | 0.94 | 3.66 | 0.96 |
| Our Story | 0.95 | 3.82 | 0.94 |
| Tip the Scale | 0.94 | 3.30 | 0.97 |
| Follow-Up 2 |  |  |  |
| Flawless | 0.89 | 3.79 | 0.78 |
| Follow-Up 3 |  |  |  |
| Flawless | 0.91 | 3.78 | 0.81 |
| Our Story | 0.92 | 3.90 | 0.79 |
| Tip the Scale | 0.92 | 3.34 | 0.89 |
| Follow-Up 4 |  |  |  |
| Let Love In | 0.91 | 3.57 | 0.82 |
| Love Me | 0.91 | 3.65 | 0.83 |
| Flawless | 0.92 | 3.84 | 0.84 |
| Our Story | 0.93 | 3.92 | 0.81 |
| Follow-Up 5 |  |  |  |
| Our Story | 0.92 | 3.94 | 0.80 |
| Love Me | 0.91 | 3.74 | 0.83 |
| Flawless | 0.91 | 3.85 | 0.82 |
| Let Love In | 0.91 | 3.56 | 0.81 |
| Follow-Up 6 |  |  |  |
| Our Story | 0.92 | 3.80 | 0.80 |
| Pick Your Poison | 0.90 | 3.64 | 0.82 |
| Labels | 0.90 | 3.62 | 0.80 |
| Love Me | 0.91 | 3.64 | 0.79 |

Rated on 1 to 5 scale, 1 = strongly disagree, 5 = strongly agree; SD = standard deviation.

**Appendix Table 4. Tobacco-Related Belief Scales and Items**

| Question | Response Options^a^ | Cronbach’s Alpha |
| --- | --- | --- |
| Health Consequences/Perceived Negative Effects | |  |
| Perceived Negative Outcomes of Occasional Smoking Scale | strongly disagree, disagree, neither agree or disagree, agree, **strongly agree** | 0.92 - 0.93 |
| If I only smoke cigarettes on some days, I will… |  |  |
| Shorten my life. |  |  |
| Damage my immune system. |  |  |
| Turn off potential partners. |  |  |
| Damage my teeth. |  |  |
| Damage my skin. |  |  |
| Develop lung cancer. |  |  |
| Perceived Negative Outcomes of Regular Smoking Scale | strongly disagree, disagree, neither agree or disagree, agree, **strongly agree** | 0.88 - 0.89 |
| If I smoke cigarettes every day, I will… |  |  |
| Shorten my life. |  |  |
| Damage my immune system. |  |  |
| Turn off potential partners. |  |  |
| Damage my teeth. |  |  |
| Damage my skin. |  |  |
| Develop lung cancer. |  |  |
| Beliefs about Living Tobacco-Free Scale (Current Smokers) | strongly disagree, disagree, neither agree or disagree, agree, **strongly agree** | 0.78 - 0.80 |
| I would be proud to live tobacco-free. |  |  |
| Living tobacco-free is important to me. |  |  |
| If I lived tobacco-free I would be proud to tell other people. |  |  |
| Using tobacco interferes with my life. |  |  |
| Tobacco use is harmful to the LGBT community. |  |  |
| Using tobacco makes life harder. |  |  |
| Individual Items |  |  |
| Smoking can cause damage to nearly every part of your body. | strongly disagree, disagree, neither agree or disagree, agree, **strongly agree** | N/A |
| Smoking weakens your immune system. |  | N/A |
| It is safe for me to smoke for only a year or two, as long as I quit after that. | **strongly disagree**, disagree, neither agree or disagree, agree, strongly agree | N/A |
| If I started to smoke occasionally, I would not become addicted. |  | N/A |
| Social Norms and Related Measures |  |  |
| Perceived Positive Attributes of Tobacco-Free People Scale | strongly disagree, disagree, neither agree or disagree, agree, **strongly agree** | 0.76 - 0.80 |
| People who are tobacco-free are… |  |  |
| Confident. |  |  |
| Trendsetting. |  |  |
| Happy. |  |  |
| Attractive. |  |  |
| Perceived Negative Attributes of Tobacco-Free People Scale | strongly agree, agree, neither agree or disagree, disagree, **strongly disagree** | 0.76 - 0.78 |
| People who are tobacco-free are… |  |  |
| Judgmental. |  |  |
| Basic. |  |  |
| Boring. |  |  |
| Predictable. |  |  |
| Injunctive Norms about Not Smoking Scale | strongly disagree, disagree, neither agree or disagree, agree, **strongly agree** | 0.82 - 0.84 |
| According to… |  |  |
| ...my family, it is very important for me to not smoke cigarettes. |  |  |
| ...most people who hang out where I hang out, it is very important for me to not smoke cigarettes. |  |  |
| ...people my age in LGBT communities it is very important for me to not smoke cigarettes. |  |  |
| Personal Rejection of Smoking in Social Situations Scale | definitely yes, probably yes, probably not, **definitely not** | 0.90 - 0.91 |
| Would you… |  |  |
| Go to a bar, club, party, concert or event where people are smoking cigarettes? |  |  |
| Hang out with someone who smokes cigarettes? |  |  |
| Dance with someone who smokes cigarettes? |  |  |
| Kiss someone who smokes cigarettes? |  |  |
| Date someone who smokes cigarettes? |  |  |
| Individual Items |  |  |
| How do LGBT people your age feel about cigarette smoking? Would you say most LGBT people your age... | **strongly disapprove**, somewhat disapprove, neither approve or disapprove, somewhat approve, strongly approve | N/A |
| Compared to 3 months ago, people my age at LGBT bars, clubs, and events are smoking… | more often, **less often**, about the same | N/A |
| Smoking Avoidance and Motivation to Quit |  |  |
| Perceived Ability to Avoid Smoking in Social Situations Scale | not at all sure, slightly sure, somewhat sure, mostly sure, **completely sure** | 0.95 |
| How sure are you that, if you really wanted to, you could avoid smoking cigarettes if… |  |  |
| You are at a party, bar or club? |  |  |
| You are in a place where most people are smoking? |  |  |
| Someone you know offers it? |  |  |
| Someone you want to get to know offers it? |  |  |
| Someone offers it to take a break? |  |  |
| Motivation/Desire to Quit Scale (Current Smokers) |  | 0.80 - 0.81 |
| How much do you want to stop smoking for good? | not at all, a little, somewhat, **a lot** |  |
| How much do you think your health would improve if you were to stop smoking for good? |  |  |
| How worried are you that smoking will damage your health in the future? | not at all worried, a little worried, somewhat worried, **very worried** |  |
| How worried are you that smoking will damage your physical appearance or attractiveness? |  |  |

^a^ Individual items were dichotomized with the bolded response option as the category of interest.

## Additional Covariate Definitions

Smoking Status is defined as five categories including:

- Never smokers (those who report never having smoked in their lifetime)
- Ever but not current smokers (those who report having smoked in their lifetime but smoked on 0 of the past 30 days)
- Non-daily “phantom” smokers (those who report having smoked on 1-29 of the past 30 days but do not consider themselves smokers)
- Non-daily smokers (those who report having smoked on 1–29 of the past 30 days and consider themselves smokers)
- Daily smokers (those who report having smoked on all 30 of the past 30 days)

Media use scales are defined as the frequency of use of TV, YouTube, Facebook, Instagram, Snapchat, Twitter, and Tumblr on 7-point scales of:

- Several times a day
- About once a day
- 3–5 days a week
- 1–2 days a week
- Every few weeks
- Less often
- Never

Lesbian, gay, bisexual, and transgender (LGBT) Community Involvement Scale is defined as the sum of dichotomized response values for:

- Have you ever attended a LGBT pride event? (0 [no] or 1 [yes])
- Have you attended a LGBT pride event in the past 12 months? (0 [no] or 1 [yes])
- In the past 30 days, on how many days did you go to an LGBT party, night, bar, club, or event? (0 [0 days] or 1 [1 day–7+ days])
- How many LGBT celebrities, athletes, musicians, or artists do you follow on social media? (0 [none] or 1 [1–5 or more])

LGBT Identity/Connection Scale contains 10 items modified from or directly from the LGBT Identity Affirmation scale (Mohr & Kendra, 2011), LGBT Identity Centrality scale (Mohr & Kendra, 2011), and Identification with the LGBT Community scale (Riggle & Mohr, 2015, Riggle et al., 2014). It was defined as the average Likert scale score (1 [strongly disagree] to 5 [strongly agree]) for the following items:

- I am glad to be an LGBT person.
- I am proud to be LGBT.
- I’m proud to be part of the LGBT community.
- My sexual identity is an insignificant part of who I am.
- My sexual identity is a central part of my identity.
- To understand who I am as a person, you have to know that I’m LGBT.
- Being an LGBT person is a very important aspect of my life.
- I believe being LGBT is an important part of me.
- I feel part of the LGBT community.
- I feel accepted by the LGBT community.

Appendix Table 5. Knowledge, Attitudes, and Beliefs (KAB) Scales with No Significant Results

| Outcome | Follow-Up Round | N^a^ | Range of DiD Estimates^b^ | Range of p-Values | Benjamini-Hochberg Corrected Significance^c^ |
| --- | --- | --- | --- | --- | --- |
| Motivation/Desire to Quit Scale (Current Smokers) | No significant effects at any follow-up | 5,270 | -0.112 to -0.013 | 0.13 to 0.86 | Not significant |
| Perceived Negative Outcomes of Regular Smoking Scale | No significant effects at any follow-up | 11,680 | -0.020 to 0.027 | 0.33 to 0.97 | Not significant |
| Personal Rejection of Smoking in Social Situations Scale | No significant effects at any follow-up | 11,671 | -0.007 to 0.029 | 0.38 to 0.99 | Not significant |
| Perceived Negative Attributes of Tobacco-Free People Scale | No significant effects at any follow-up | 11,669 | -0.032 to 0.025 | 0.44 to 0.89 | Not significant |
| Injunctive Norms about Not Smoking Scale | No significant effects at any follow-up | 11,668 | -0.026 to 0.029 | 0.53 to 0.99 | Not significant |
| Perceived Negative Outcomes of Occasional Smoking Scale | No significant effects at any follow-up | 11,670 | -0.007 to 0.025 | 0.53 to 0.96 | Not significant |
| Beliefs about Living Tobacco-Free Scale (Current Smokers) | No significant effects at any follow-up | 5,281 | -0.007 to 0.030 | 0.60 to 0.98 | Not significant |

^a^ N is the number of unique respondents in each model, with between 1 and 7 observations per respondent.

^b^ Difference-in-difference (DiD) estimate is the contrast between the change in predicted scores from baseline to a given follow-up round for Treatment vs. Control. In cases where the outcome had no significant results, the range of DiD estimates (and corresponding p-values) is reported.

^c^ All p-values were adjusted for multiple comparisons using a false discovery rate of 20%.

Note: Control variables in linear regression models with fixed effects were age, education, employment status, student status, LGBT identity, smoking status, and scales for media use, LGBT involvement, and LGBT connection.

CI = confidence interval

Appendix Table 6. KAB Scale Significant Results by Smoking Status Subgroups

| Outcome | Subgroup | Follow-Up Round | N^a^ | DiD Estimate [95% CI] or Range of Estimates^b^ | p-Value | Benjamini-Hochberg Corrected Significance^c^ |
| --- | --- | --- | --- | --- | --- | --- |
| Perceived Positive Attributes of Tobacco-Free People Scale | Never smokers | Follow-up 2 Follow-up 3 | 11,667 | 0.178 [0.021, 0.335] 0.176 [0.024, 0.327] | 0.03 0.02 | Not significant Not significant |
|  | Ever not current smokers | Follow-up 5 |  | 0.130 [0.003, 0.257] | 0.04 | Not significant |
|  | Non-daily smokers | Follow-up 1 |  | 0.290 [0.014, 0.566] | 0.04 | Not significant |
| Perceived Ability to Avoid Smoking in Social Situations Scale | Never smokers | Follow-up 4 | 11,677 | 0.094 [0.003, 0.186] | 0.04 | Not significant |
|  | Ever not current smokers | Follow-up 6 |  | 0.124 [0.005, 0.244] | 0.04 | Not significant |
|  | Non-daily phantom smokers | Follow-up 6 |  | 0.303 [0.071, 0.535] | 0.01 | Not significant |
| Motivation/Desire to Quit Scale (Current Smokers) | Non-daily phantom smokers | Follow-up 1 | 5,270 | −0.202 [−0.398, −0.007] | 0.04 | Not significant |
|  | Daily smokers | Follow-up 5 |  | −0.270 [−0.501, −0.039] | 0.02 | Not significant |
| Perceived Negative Outcomes of Regular Smoking Scale | Non-daily phantom smokers | Follow-up 1 | 11,680 | −0.142 [−0.272, −0.012] | 0.03 | Not significant |
| Personal Rejection of Smoking in Social Situations Scale | Non-daily phantom smokers | Follow-up 1 | 11,671 | −0.141 [−0.271, −0.012] | 0.03 | Not significant |
|  | Daily smokers | Follow-up 6 |  | 0.242 [0.036, 0.448] | 0.02 | Not significant |
| Perceived Negative Attributes of Tobacco-Free People Scale | Daily smokers | Follow-up 2 | 11,669 | 0.362 [0.110, 0.613] | < 0.01 | Not significant |
| Injunctive Norms about Not Smoking Scale | Ever not current smokers | Follow-up 3 | 11,668 | 0.168 [0.018, 0.318] | 0.03 | Not significant |
|  | Non-daily phantom smokers | Follow-up 3 Follow-up 5 |  | −0.182 [−0.359, −0.004] −0.215 [−0.408, −0.022] | 0.04 0.03 | Not significant  Not significant |
|  | Daily smokers | Follow-up 2 |  | −0.395 [−0.688, −0.103] | < 0.01 | Not significant |
| Perceived Negative Outcomes of Occasional Smoking Scale | No significant subgroup results at any follow-up | | 11,670 | −0.156 to 0.238 | 0.09 to 0.94 | Not significant |
| Beliefs about Living Tobacco-Free Scale (Current Smokers) | Daily smokers | Follow-up 1 | 5,281 | 0.258 [0.030, 0.487] | 0.03 | Not significant |

^a^ N is the number of unique respondents in each model, with between 1 and 7 observations per respondent.

^b^ Difference-in-differences (DiD) estimate is the contrast between the change in predicted scores from baseline to a given follow-up round for Treatment vs. Control. In cases where the outcome had no significant results, the range of DiD estimates (and corresponding p-values) is reported.

^c^ All p-values were adjusted for multiple comparisons using a false discovery rate of 20%.

Note: Control variables in linear regression models with fixed effects were age, education, employment status, student status, LGBT identity, smoking status, and scales for media use, LGBT involvement, and LGBT connection.

CI = confidence interval; LGBT = lesbian, gay, bisexual, and transgender.

Appendix Table 7. Individual Knowledge, Attitude, and Belief (KAB) Items with No Significant Results

| **Outcome** | **Follow-Up Round** | N^a^ | **Range of DiD Estimates^b^** | **Range of p-Values** | **Benjamini-Hochberg Corrected Significance^c^** |
| --- | --- | --- | --- | --- | --- |
| Tobacco use is harmful to the LGBT community. | No significant effects at any follow-up | 11,652 | −0.005 to 0.037 | 0.06 to 0.78 | Not significant |
| If I only smoke cigarettes on some days (for example, smoking only when I go out), I will turn off potential partners. | No significant effects at any follow-up | 11,655 | −0.034 to 0.010 | 0.07 to 0.67 | Not significant |
| How sure are you that, if you really wanted to, you could avoid smoking cigarettes if you are in a place where most people are smoking? | No significant effects at any follow-up | 11,673 | 0.006 to 0.033 | 0.07 to 0.73 | Not significant |
| Compared to 3 months ago, people my age at LGBT bars, clubs, and events are smoking… | No significant effects at any follow-up | 10,316 | −0.004 to 0.033 | 0.08 to 0.85 | Not significant |
| Would you date someone who smokes cigarettes? | No significant effects at any follow-up | 11,623 | 0.002 to 0.025 | 0.10 to 0.92 | Not significant |
| How much do you want to stop smoking for good? | No significant effects at any follow-up | 5,144 | −0.050 to 0.012 | 0.10 to 0.80 | Not significant |
| It is safe for me to smoke for only a year or two, as long as I quit after that. | No significant effects at any follow-up | 11,648 | −0.031 to 0.014 | 0.11 to 0.56 | Not significant |
| How do LGBT people your age feel about cigarette smoking? | No significant effects at any follow-up | 11,615 | −0.011 to 0.015 | 0.11 to 0.62 | Not significant |
| If I only smoke cigarettes on some days (for example, smoking only when I go out), I will damage my immune system. | No significant effects at any follow-up | 11,650 | 0.002 to 0.029 | 0.11 to 0.90 | Not significant |
| How sure are you that, if you really wanted to, you could avoid smoking cigarettes if someone you want to get to know offers it? | No significant effects at any follow-up | 11,664 | −0.034 to 0.011 | 0.11 to 0.90 | Not significant |
| How worried are you that smoking will damage your health in the future? | No significant effects at any follow-up | 5,249 | −0.022 to 0.044 | 0.14 to 0.80 | Not significant |
| Would you go to a bar, club, party, concert or event where people are smoking cigarettes? | No significant effects at any follow-up | 11,645 | −0.010 to 0.011 | 0.14 to 0.91 | Not significant |
| How much do you think your health would improve if you were to stop smoking for good? | No significant effects at any follow-up | 5,228 | −0.035 to 0.048 | 0.15 to 0.54 | Not significant |
| How sure are you that, if you really wanted to, you could avoid smoking cigarettes if someone offers it to take a break? | No significant effects at any follow-up | 11,672 | −0.009 to 0.026 | 0.15 to 0.94 | Not significant |
| Smoking weakens your immune system. | No significant effects at any follow-up | 11,660 | −0.028 to 0.001 | 0.17 to 0.94 | Not significant |
| Smoking can cause damage to nearly every part of your body. | No significant effects at any follow-up | 11,660 | −0.028 to 0.011 | 0.20 to 0.87 | Not significant |
| If I smoke cigarettes every day, I will turn off potential partners. | No significant effects at any follow-up | 11,661 | −0.025 to 0.021 | 0.21 to 0.75 | Not significant |
| If I only smoke cigarettes on some days (for example, smoking only when I go out), I will develop lung cancer. | No significant effects at any follow-up | 11,643 | −0.001 to 0.023 | 0.21 to 1.00 | Not significant |
| According to my family, it is very important for me to not smoke cigarettes. | No significant effects at any follow-up | 11,657 | −0.010 to 0.023 | 0.24 to 0.96 | Not significant |
| If I only smoke cigarettes on some days (for example, smoking only when I go out), I will damage my skin. | No significant effects at any follow-up | 11,655 | −0.019 to 0.008 | 0.31 to 0.99 | Not significant |
| How sure are you that, if you really wanted to, you could avoid smoking cigarettes if someone you know offers it? | No significant effects at any follow-up | 11,672 | −0.008 to 0.016 | 0.38 to 0.90 | Not significant |
| If I only smoke cigarettes on some days (for example, smoking only when I go out), I will damage my teeth. | No significant effects at any follow-up | 11,657 | −0.017 to 0.012 | 0.38 to 0.98 | Not significant |
| If I only smoke cigarettes on some days (for example, smoking only when I go out), I will shorten my life. | No significant effects at any follow-up | 11,651 | 0.0004 to 0.015 | 0.42 to 0.98 | Not significant |

^a^ N is the number of unique respondents in each model, with between 1 and 7 observations per respondent.

^b^ Difference-in-differences (DiD) estimate is the contrast between the change in predicted probabilities from baseline to a given follow-up round for Treatment vs. Control. In cases where the outcome had no significant results, the range of DiD estimates (and corresponding p-values) is reported.

^c^ All p-values were adjusted for multiple comparisons using a false discovery rate of 20%.

Note: Control variables in logistic regression models with random effects were age, education, employment status, student status, smoking status, LGBT identity, race/ethnicity, recruitment source, and scales for media use, LGBT involvement, and LGBT connection.

CI = confidence interval; LGBT = lesbian, gay, bisexual, and transgender.
